# From Cortical and Subcortical Grey Matter Abnormalities to Neurobehavioral Phenotype of Angelman Syndrome: A Voxel-Based Morphometry Study

**DOI:** 10.1371/journal.pone.0162817

**Published:** 2016-09-14

**Authors:** Gayane Aghakhanyan, Paolo Bonanni, Giovanna Randazzo, Sara Nappi, Federica Tessarotto, Lara De Martin, Francesca Frijia, Daniele De Marchi, Francesco De Masi, Beate Kuppers, Francesco Lombardo, Davide Caramella, Domenico Montanaro

**Affiliations:** 1 Unit of Neuroradiology, Fondazione CNR/Regione Toscana G. Monasterio, Pisa, Italy; 2 Epilepsy and Clinical Neurophysiology Unit, E. Medea Scientific Institute, Conegliano (TV), Italy; 3 Division of Anesthesiology and Intensive Care, University Hospital of Pisa, Pisa, Italy; 4 Diagnostic and Interventional Radiology, Department of Translational Research and New Technologies in Medicine and Surgery, University of Pisa, Pisa, Italy; Alberta Children's Hospital, CANADA

## Abstract

Angelman syndrome (AS) is a rare neurogenetic disorder due to loss of expression of maternal ubiquitin-protein ligase E3A (UBE3A) gene. It is characterized by severe developmental delay, speech impairment, movement or balance disorder and typical behavioral uniqueness. Affected individuals show normal magnetic resonance imaging (MRI) findings, although mild dysmyelination may be observed. In this study, we adopted a quantitative MRI analysis with voxel-based morphometry (FSL-VBM) method to investigate disease-related changes in the cortical/subcortical grey matter (GM) structures. Since 2006 to 2013 twenty-six AS patients were assessed by our multidisciplinary team. From those, sixteen AS children with confirmed maternal 15q11-q13 deletions (mean age 7.7 ± 3.6 years) and twenty-one age-matched controls were recruited. The developmental delay and motor dysfunction were assessed using Bayley III and Gross Motor Function Measure (GMFM). Principal component analysis (PCA) was applied to the clinical and neuropsychological datasets. High-resolution T1-weighted images were acquired and FSL-VBM approach was applied to investigate differences in the local GM volume and to correlate clinical and neuropsychological changes in the regional distribution of GM. We found bilateral GM volume loss in AS compared to control children in the striatum, limbic structures, insular and orbitofrontal cortices. Voxel-wise correlation analysis with the principal components of the PCA output revealed a strong relationship with GM volume in the superior parietal lobule and precuneus on the left hemisphere. The anatomical distribution of cortical/subcortical GM changes plausibly related to several clinical features of the disease and may provide an important morphological underpinning for clinical and neurobehavioral symptoms in children with AS.

## Introduction

Angelman syndrome (AS) is a rare neurogenetic disorder (Online Mendelian Inheritance in Man, OMIM 105830) with estimation of one in 12,000–20,000 population [1,2]. Despite of low prevalence, it is the most studied human diseases related to a genomic imprinting—a process that determines the differential expression of genes according to their maternal or paternal origin [3]. The AS results from the loss of maternally inherited ubiquitin-protein ligase E3A gene (UBE3A) in the chromosomal 15q11.q13 region, which exhibits imprinting in the central nervous system. The UBE3A gene encodes for the E3A ubiquitin ligase that targets short-lived and abnormal proteins for ubiquitination and has varied expression in different brain regions [4]. The mouse models of AS suggest that the paternal UBE3A expression is epigenetically silenced (imprinted) in some brain regions, such as the hippocampus, cerebellar Purkinje cells and olfactory bulb [5]. Therefore, the maternal inactivation of UBE3A in AS causes a nearly complete loss of UBE3A protein from these selective brain regions [6]. In addition, various areas of the brain like cortex, striatum, midbrain and hypothalamus showed predominant expression from the maternal copy of the chromosome, and as a consequence, these regions express larger reduction in the levels of UBE3A in AS [7,8].

In the majority of cases, AS arises from deletions within chromosome 15q11-q13 (75% of cases) and intragenic mutation (15% of cases), while a minority of AS cases arise from microdeletions that affect imprinting at the 15q11-q13 locus (2–4%) or from paternal uniparental disomy (PUD) (7%) [9].

Children with AS have apparently normal phenotype at early stages of infancy. Developmental delay is first noted at around age of six months, but the unique clinical features of AS do not manifest until the first year of life. The consistent clinical characteristics that are present in all AS individuals are the severe developmental delay, speech impairment, movement or balance disorder with tremulousness of limbs, and typical behavioral uniqueness including frequent smiling, happy demeanour and hyper-motor behavior [10]. Frequent clinical findings (more than 80%) are microcephaly, epileptic seizures and abnormal electroencephalography (EEG). Up to 20–80% of AS patients may be presented with associated clinical features, such as facial dysmorphism, strabismus, protruding tongue, frequent drooling, excessive chewing behaviors, obesity in older children, characteristic wide-based gait, sleep disorders and scoliosis [10,11].

The pathogenesis of AS has been largely studied in the mouse models of AS, and in particular, the UBE3A-maternal knockout mice was extensively used [12]. This model produces phenotypes with characteristic motor and cognitive features resembling AS. It provided enormous insights in the understanding of the disease molecular mechanism and consolidated the fact that a primarily responsible cause for AS is the loss of expression of maternal-inherited UBE3A [8]. Yet, the open challenge persists in identifying the neural target substrates of UBE3A. While overall brain morphology and connectivity of neural projections appear largely normal in the mouse models of AS, the major functional defects are detected at the level of context-dependent learning in line with impaired maturation of hippocampal and neocortical circuits [8,13]. These findings demonstrate a crucial role for UBE3A in synaptic development, function, and plasticity [14,15]. Furthermore, the lack of regional selective expression of UBE3A may exhibit distinct fingerprint on the functional and structural organization of the brain in AS.

Magnetic resonance imaging (MRI) and advance neuroimaging techniques can contribute to understanding of the severe neurobehavioral phenotype of AS, however the paucity of neuroimaging studies reported in the scientific literature is quite surprising. Standard brain MRI may show minor abnormalities, such as mild cortical atrophy, dysmyelination and focal white matter signal abnormalities [16,17]. A small number of studies applying advance neuroimaging approaches, such as diffusion tensor imaging and tractography, reported global impairment of white matter (WM) integrity and aberrant connectivity in temporal white matter pathways associated with language and cognitive functioning in the brain of AS patients [18–20]. Meanwhile, the cortical and subcortical grey matter (GM) structures were not yet studied with the means of computational techniques, even though the animal research denotes those structures for their possible involvement in the pathophysiology of AS [7,8]. Therefore, we adopted a quantitative voxel-based morphometry (FSL-VBM) method to investigate disease-related changes in the cortical/subcortical GM structures and to search for morphometric representation of AS phenotype. By using a whole brain analysis, VBM does not require any a priori assumption about the location of possible differences and offers a relatively unbiased, operator-independent method for searching brain structural changes [21].

## Material and Methods

### Participants

Since 2006 and 2013 twenty-six patients with clinically and genetically documented AS were assessed at the Unit of Epilepsy and Clinical Neurophysiology of the IRCCS Medea, in Conegliano (TV), Italy. They are reviewed systematically at regular intervals by the same multidisciplinary team, including a pediatric neurologist, neuroradiologist, physiatrist, ophthalmologist, pediatrician, physiotherapist, psychologist, speech therapist and consultant pediatric orthopedic surgeon. Diagnostic workflow included video-polygraphic-EEG recording during both wakefulness and sleep, long-term video-EEG monitoring in patients with seizure progression and brain MRI examination for excluding other causes of epilepsy [22].

All AS patients were screened with fluorescence in situ hybridization and DNA methylation tests. Twenty-one patients presented deletions of 15q11-13, four patients had confirmed PUD and one patient showed UBE3A mutation. For the purpose of this study, in order to avoid the attribution of potentially confounding genetic factors, we included only AS patients with deletions of the maternally inherited UBE3A gene located in the chromosome 15q11-q13. Four out of twenty-one patients fell out of the age distribution of the cohort (< 3.5 years and >15 years). One patient has been excluded due to structural brain abnormalities (bilateral polymicrogyria). Overall, sixteen AS children with maternal 15q11-q13 deletions (mean age 7.7 ± 3.6 years, eleven males) and twenty-one age-matched controls (mean age 8.5 ± 3.3 years, eleven males) were recruited for this study.

Control subjects were normal developing children enrolled from the routine clinical MRI waiting list and have assigned to brain MRI examination due to unrelated causes (e.g. mild/moderate unilateral conductive hypoacusis and headache disorder). Children included in the control group: (a) had an absence of any neurological or psychiatric diagnoses, (b) were not taking any regular medications, (c) had no clinical elevations on a caregiver-reported measure of behavioral problems.

Written informed consent was obtained from parents or caregivers on the basis of legal guardians for AS and control subjects. The study was designed in accordance with the ethical standards of the Declaration of Helsinki (revision of 2008) and approved by the Ethics Committee of the Azienda Ospedaliero-Universitaria Pisana (protocol number 3255, approved on 20/01/2009).

### Clinical and neuropsychological evaluation

#### Clinical assessment

Angelman syndrome was clinically evaluated using the diagnostic criteria of Williams et al [10]. The movement or balance disorder was classified by performance and considered to be present when there was an uncoordinated movement pattern comprising broad-based gait, stiff lower limbs on ambulation, jerky motor function, and a disturbed sense of equilibrium in the upright position with compensating arm movements. The presence of tremor was observed both at rest and while performing the fine motor function during clinical and neurophysiological tests for each participant. The tremor was classified as “cortical myoclonus (CM)” if there were EEG changes [23], or “postural and action tremor (AT)” (nonepileptic) if there weren’t EEG changes [23,24]. Concomitant psychotropic and anti-epileptic medications included (number of subjects in parentheses): Risperidone (2), Valproate (15), Levetiracetam (1), Clonazepam (6), Ethosuximide (7), Clobazam (2), Melatonin (8). No patient received sex hormone replacement or growth hormone therapy.

### Bayley scales of infant and toddler development

To quantify developmental delay, AS patient were assessed using the Bayley Scales of Infant and Toddler Development, 3rd edition (BSID-III) [25]. Although the BSID-III is standardized for children with a chronological age between 1 and 42 months, it is also used for children beyond that normative age range because children with developmental disabilities should not be assessed using instruments that are appropriate for their chronological age [26,27]. The Bayley III comprises 5 scales: cognitive, language (expressive and receptive) and motor (gross and fine). Developmental delay in percentage was calculated according to the Bayley-III norm [28] by dividing the raw scores of the developmental age equivalents (DA) by the chronological age (CA) in months, converting the resulting decimal quotient into a percentage and subtracting that percentage from 100%.

### Gross motor function measure

To quantify motor dysfunction, our patients were assessed using the Gross Motor Function Measure (GMFM), which is clinically validated test designed to evaluate changes in gross motor functions in children with cerebral palsy [29]. We used the GMFM 88 items version (GMFM-88), which includes several simple tasks performed in five dimensions: 1) lying-rolling, 2) sitting, 3) creeping-kneeling, 4) standing, 5) walking-running-jumping. The GMFM-88 has been already reported to be valid measures in patients with AS syndrome [30,31]. Quantification is based on how much of the task the patient can realize independently, without any reference to the quality of the performance. The GMFM-88 item scores are then summed to calculate raw and percent scores for each of the five GMFM dimensions.

### Imaging protocol

Children with AS received sedation according to the institutional standard sedation protocol administered by trained anesthesiologists to achieve the degree of cooperation and immobilization required to successfully complete the MRI examination. Control children underwent an MRI examination according to the protocol they have been directed to. Each subject’s MRI scan was evaluated by two experienced neuroradiologists (DM, FL) for gross anatomical abnormalities and all datasets were assessed for a quality control.

### MRI acquisition

All patients and controls underwent MRI brain scans with 3.0 T system (GE Excite HDx, GE Medical Systems, Wisconsin, Milwaukee, USA) at the Foundation CNR/Regione Toscana G. Monasterio, Pisa, Italy. A T1-weighted Spoiled Gradient Recalled Acquisition (SPGR) sequence generated 160 contiguous axial slices (TR/ TE = 10.7/4.9 msec, FOV: 25.6cm, acquisition matrix: 256x256, thickness: 1mm, slice gap: 0mm, BW: 15.6kHz, NEX: 1) used to obtain high resolution three-dimensional images.

#### MRI score

We used a simple scoring system developed in this study to assess gross structural abnormalities in the brain. The WM abnormalities were graded according to 4 scales and assess the extent of incomplete myelination or other WM signal abnormalities (0-normal; 1—mild; 2—moderate; 3—severe abnormalities). The subarachnoidal spaces were graded according to 3 scales (0-normal; 1—focally enlarged and 2—diffusely enlarged). The ventricular system was graded according to 4 scales (0—normal; 1—dilatation of the lateral ventricles; 2—predominantly supratentorial dilatation; 3—diffuse dilatation). Cortical and cystic abnormalities were graded according to yes/no scale. All scans were scored independently by two neuroradiologists (DM, FL).

#### Quantitative image post-processing

Total brain tissue volume, normalised for subject head size, was estimated with SIENAX (http://fsl.fmrib.ox.ac.uk/fsl/fslwiki/SIENA), part of FSL [32]. SIENAX starts by extracting brain and skull images. The first is affine-registered to MNI152 space, while the second is used to determine the registration scaling for head size normalisation. It then runs tissue segmentation into different tissue types (GM, WM and cerebrospinal fluid, CSF) to estimate the volume of brain tissue, and multiplies this by the estimated scaling factor, to reduce head-size-related variability between subjects [33].

To investigate voxel-wise differences in the local grey matter volume/topography we used FSL-VBM [21], an optimized VBM protocol [34] carried out with FSL tools [32] (http://fsl.fmrib.ox.ac.uk/fsl/fslwiki/). First, structural images were brain-extracted and GM segmented before being registered to the MNI152 standard space using non-linear registration [35]. The resulting images were averaged and flipped along the x-axis to create a left-right symmetric, study-specific GM template. Second, all native GM images were non-linearly registered to this study-specific template and "modulated" to correct for local expansion (or contraction) caused by the non-linear component of the spatial transformation. The modulated GM images were then smoothed with an isotropic Gaussian kernel with a sigma of 3 mm.

### Statistical analysis

Descriptive statistics were calculated for demographic, clinical, and neuropsychological measures. Inter-rater reliability of category assignment and weighted kappa coefficient has been used for correlation of two raters. Between-group differences were assessed using non-parametric Mann-Whitney U-tests for continuous variables and Pearson Chi-square test for categorical variables. Spearman’s method was used for correlation analysis of relationships between continuous variables. The alpha-level less than 0.05 was set to determine the significance.

#### Principal components analysis

Principal component analysis (PCA) was applied to reduce the dimensionality of the complex clinical and neuropsychological dataset consisting variables that are highly correlated with each other. The PCA produces linear combinations of covariates transforming the observed variables into lower dimensional compounds, principal components (PC), that are uncorrelated and explain the variation in the data. No rotation was carried out to the PCA solution preserving its optimality properties. The PCs with eigenvalue close or higher to 2 were extracted. An arbitrary cut-off of 0.3 was used to determine whether a variable was loaded onto the respective PC.

Analyses were performed by using R software (http://www.r-project.org/).

#### Voxel-wise statistics

Design matrix and contrast files were generated using the FMRIB Software Library General Linear Model Graphical User Interface. Between-group differences in GM volume were carried out with unpaired voxel-wise t-tests within the framework of the general linear model (GLM) accounting age, gender and WM volume as a nuisance covariate. Since the FSL-VBM pipeline removes any variance due to differences in head size, the total intracranial volume does not need to be included as a confounding covariate, hence, we have incorporated the differences due to age, gender and WM atrophy pattern [36]. The GLM was also used to test the voxel-wise effect of the neuropsychological measures and PCs (PCA output) on the volume by conducting a single group model with additional covariates. Statistical inference was carried out using permutation-based non-parametric testing with 5000 permutations with threshold-free cluster enhancement (TFCE) option (Randomise, part of the FSL tools), which is an optimized method to detect clusters without having to define clusters in a binary way [37]. Multiple comparisons across space were controlled and family-wise error rate corrected p-values less than 0.05 were accepted.

#### Cluster volume calculation

To obtaining regional GM volume within the significant cluster after applying between-group voxel-wise VBM analysis, we, first, thresholded the t-map at p > 0.05, corrected for multiple comparisons, then binarized and masked it using the fslmaths program (part of FSL, http://fsl.fmrib.ox.ac.uk/fsl/fslwiki/Fslutils), which allows mathematic manipulation of the images. Than, we used the fslstats program (part of FSL, http://fsl.fmrib.ox.ac.uk/fsl/fslwiki/Fslutils), to calculate the GM volume within the mask for each subject.

## Results

Children of the AS and control groups did not differ in terms of age and gender distribution. The demographic, clinical/radiological and neuropsychological profiles are summarized in Table 1.

**Table 1:** Demographic and clinical/radiological profiles of children with Angelman syndrome and controls.

		Angelman *N = 16*	Control *N = 21*	P-values
Age	*years*	7.9 (8.5 ± 3.3)	6.6 (7.7 ± 3.6)	0.31^1^
Gender: Male		52% (11)	69% (11)	0.32^2^
MRI score		2.9 (1.9 ± 3)	0	< 0.001^1^
Ventricular CSF	*cm*^*3*^	27.4 (33.2 ± 18.6)	21.1 (22.3 ± 8.1)	0.038^1^
Total GM	*cm*^*3*^	930 (947 ± 81)	989 (1015 ± 77)	0.008^1^
Total WM	*cm*^*3*^	628 (635 ± 50)	707 (712 ± 33)	< 0.001^1^
Total brain	*cm*^*3*^	1579 (1585 ± 111)	1720 (1726 ± 82)	< 0.001^1^
Volume of GM cluster	*cm*^*3*^	16.2 (16.6 ± 1.1)	21.5 (22.3 ± 1.9)	< 0.001^1^
Delay sitting	*months*	7.5 (13.0 ± 11.2)	-	-
Delay walking	*years*	2.9 (3.5 ± 1.9)	-	-
Seizure onset	*months*	26.5 (27.6 ± 11.2)	-	-
**Bayley III**				
• cognitive	*months*	16.5 (15.56 ± 5.14)	-	
• expressive communication	*months*	12 (11.12 ± 4.53)	-	
• receptive communication	*months*	17 (15.75 ± 5.32)	-	-
• fine motor	*months*	12.5 (13.25 ± 5.45)	-	-
• gross motor	*months*	17.25 (14.66 ± 4.5)	-	-
**GMFM**				
• lying and rolling	*%*	100 (92.65 ± 11.5)	-	-
• sitting	*%*	85 (80 ± 18.9)	-	-
• crawling and kneeling	*%*	46.4 (40.2 ± 32.4)	-	-
• standing	*%*	55.1 (46.3 ± 27.9)	-	-
• walking, running and jumping	*%*	29.2 (30.56 ± 20.5)	-	-

Abbreviations: GM, grey matter; GMFM, gross motor function measure; CSF, cerebrospinal fluid; MRI, magnetic resonance imaging, WM, white matter; a(x ± s) represents Median (Mean ± 1 SD). Numbers after percents are frequencies.

Tests used:

^1^ Wilcoxon test;

^2^ Pearson test

All patients presented with the classical AS deletion phenotype. The consistent clinical characteristics were the severe developmental delay, speech impairment, movement or balance disorder and typical behavioral including happy demeanor and hype-rmotor behavior. Tremulousness of limbs was present in 13 patients (82%), of these 9 had both CM and (nonepileptic) AT, and 4 only AT and no patients had CM only. Epilepsy was present in 15 patients (94%) and was characterized by focal (versive) seizures (80%), atypical absences (66.5%), secondarily generalized seizures (26.5%) and status epilepticus (67%), convulsive in 27% and nonconvulsive in 40%. Eight patients presented more than 1 type of seizure (53%). Inter-rater agreement for the summary MRI score was 73% (weighted kappa = 0.93, p < 0.001).

### Principal components analysis

The temporal step of the PCA resulted in overall 15 PCs, which are the underlying structure of the data, the directions where there is the most variance and where the data is most spread out. We extracted the first three principal components (p = 3) that explain over 75% of the total variability in the standardized ratings. Hence, the original 15-dimensional clinical and neuropsychological data was condensed by PCA into a three compounds. The PC1 mainly represented the Bayley tests scores, including cognitive, language and motor skills; the PC2 condensed GMFM test scores, CA and seizure onset; and the PC3 included the MRI score, delay in walking and GMFM walking, running and jumping scores. Initial eigenvalues indicated that the first three PCs explained 39.2%, 23.1%, and 13.2% of the variance respectively (Table 2).

**Table 2:** The coefficients for the first three principle components and the variance explained.

	PC 1	PC 2	PC 3
Delay sitting	**-0.3**	-0.13	
Delay walking	-0.12	0.19	**0.49**
Seizure onset	0.13	**-0.33**	**-0.35**
MRI score		-0.11	**0.3**
CA		**0.46**	0.11
Bayley III cognitive	**-0.37**	0.14	-0.19
Bayley III expressive communication	**-0.3**	0.13	**-0.31**
Bayley III receptive communication	**-0.3**	**0.3**	0.16
Bayley III fine motor	**-0.3**	0.11	-0.18
Bayley III gross motor	**-0.36**	0.19	-0.13
GMFM lying and rolling	0.16	**0.35**	0.24
GMFM sitting	0.28	**0.31**	-0.14
GMFM crawling and kneeling	**0.32**	0.11	0.11
GMFM standing	0.26	**0.34**	-0.23
GMFM walking, running and jumping	0.18	**0.31**	**-0.44**
% Variance explained by each PC	39.19	23.1	13.18
Cumulative % of variance explained by each PC	39.19	62.29	75.47

Abbreviations: CA, chronological age; GMFM, gross motor function measure; MRI, magnetic resonance imaging; PC, principal component. Note: Coefficients < 0.1 were suppressed to simplify the table. A cut-off of 0.3 was used to determine variables weightings onto the respective PC (emphasized with bold).

**Fig 1:**
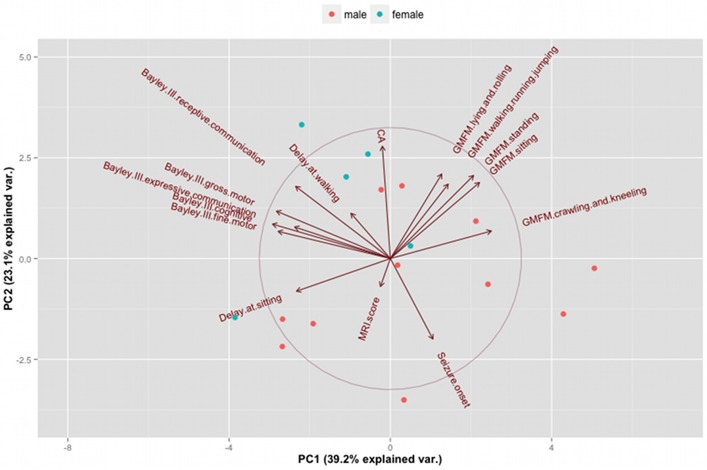
The two-dimensional biplot shows the projection of the data on the first two PCs. It colors each point according to the gender and shows the loading of each variable on the first two principal components with a circle of correlation.

The two-dimensional biplot (Fig 1) shows the projection of the data on the first two PCs.

### SIENAX output

**Fig 2:**
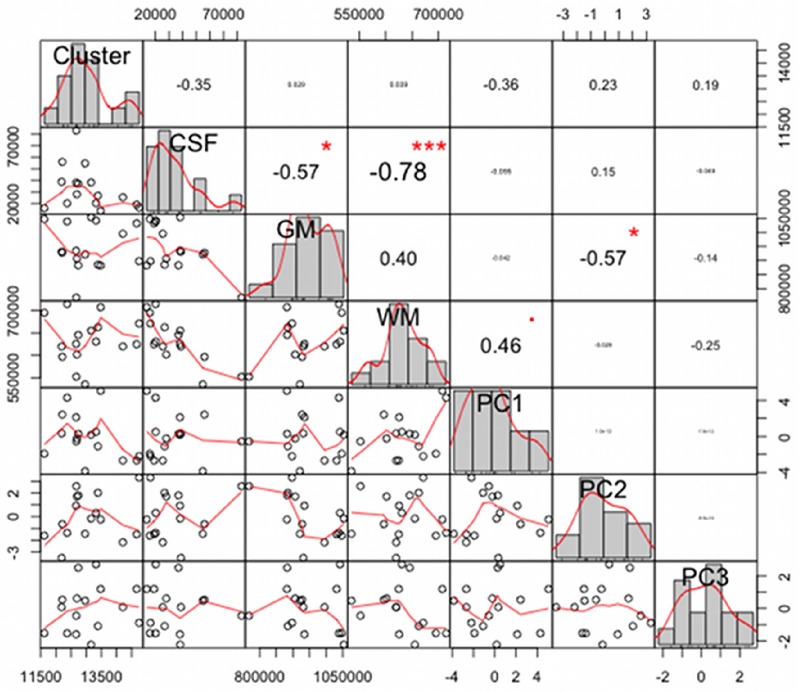
A chart of the correlation matrix. The distribution of each variable is shown on the diagonal. On the bottom of the diagonal: the bivariate scatter plots with a fitted line are displayed. On the top of the diagonal: the value of the correlation plus the significance level as stars. Abbreviations: CSF, cerebrospinal fluid; GM, grey matter; WM, white matter; PC, principal component.

The normalized brain volume, the GM/WM volumes were smaller and ventricular CSF was higher (p < 0.05) in the AS group than those in the control group (Table 1). To determine the relationship between quantitative MRI variables and first three PCs, we created the correlation matrix illustrated in the Fig 2. It shows negative strong correlation between ventricular CSF and WM (r = −0.78, p < 0.05), as well as with GM (r = −0.57, p < 0.05). Noticeably, we found significant relationship between PC2 and GM (r = −0.57, p < 0.05), while PC1 demonstrates moderate and marginal significant correlation with WM (r = −0.46, p = 0.07).

### Between-group VBM results

**Fig 3:**
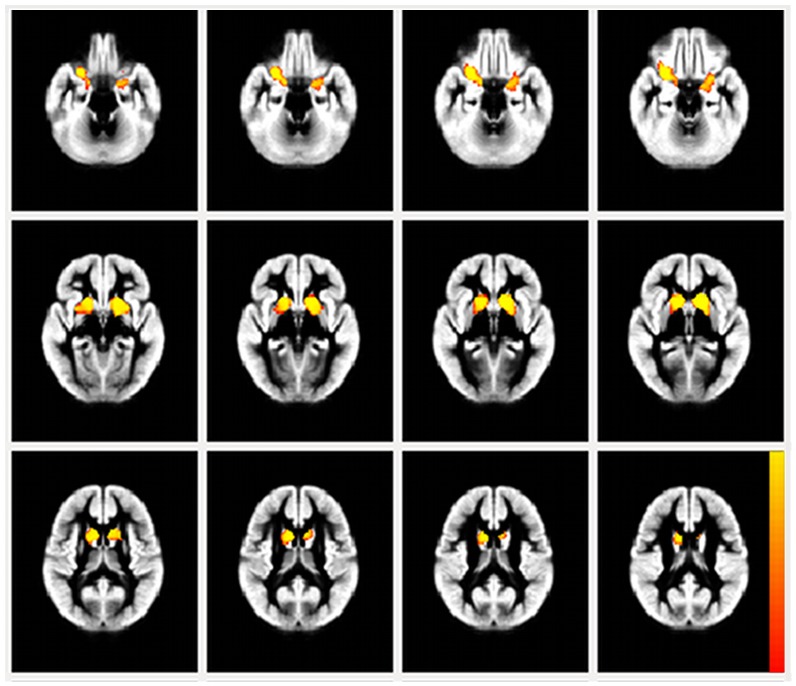
Between-group VBM analysis: the statistical map is overlaid on the study-specific grey matter template (created by FSL-VBM protocol) on axial plane. Red-yellow clusters show grey matter volumetric reduction in children with Angelman syndrome compared to controls.

Whole-brain VBM analysis of the GM volume between AS and control groups is represented in Fig 3. The AS children demonstrated smaller volume (p < 0.05, corrected) of the left and right striatum, including most of the dorsal striatum (putamen, caudate head, and body). Both right and left clusters extended to the temporal-mesial structures, including the amygdala, hippocampus and parahippocampal gyrus. The right cluster is largely broadened to the orbitofrontal cortex (OFC) and partially to insular cortex.

Table 3 provides an overview of each cluster characteristics (cluster size, MNI coordinates for location of maximum, labeled anatomical structures and the probability values).

**Table 3:** Size and labelled anatomical structures of the significant clusters (p < 0.05, corrected). The voxel coordinates (in millimeter) in MNI standard space for the location of the maximum intensity are presented. The probability values are scaled from 0 to 100 and indicate the probability of the cluster being a member of the different labelled regions within the atlas.

Cluster index	Voxels	Location ofmaximum			Anatomical structures	Probabiliy (%)
		x	y	z		
1	1840	28	12	-26	caudate right	19.1
					putamen right	12.9
					pallidum right	2.3
					amygdala right	2.3
					accumbens right	1.8
					insular cortex, right	2.8
					temporal pole, right	2.5
					frontal orbital cortex, right	11.1
					parahippocampal gyrus, right	2.7
2	1737	-14	14	-2	caudate left	17.6
					putamen left	16.4
					pallidum left	3.04
					amygdala left	11.1
					accumbens left	3.4
					frontal orbital cortex, left	2.1
					parahippocampal gyrus, left	2.1

We launched various correlation analyses between the mean GM volumes calculated for each subject within the mask of the significant cluster and the clinical/neuropsychological scores, as well as with the first three PCs, but no significant relationship was found (Fig 2).

### Single group averages with additional covariates

**Fig 4:**
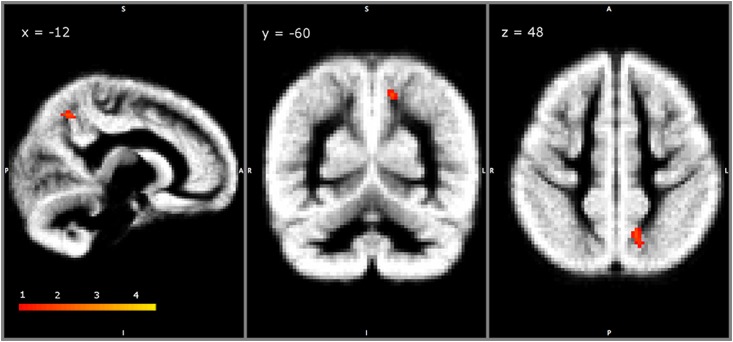
Single group VBM analysis with additional covariates (PCs): the statistical map represents the correlation between PC2 and GM volume in AS children overlaid on the study-specific grey matter template (created by FSL-VBM protocol) on the sagittal, coronal and axial plane.

No voxel-wise relationship was found between GM topography of AS children and a) Bayley III DA equivalent scores in cognitive, language (expressive and receptive) and motor (gross and fine) domains; as well as with b) GMFM summary score, or with each GMFM item separately (lying and rolling; sitting; crawling and kneeling; standing; walking, running and jumping). Considering that our statistical model might suffer from high correlation between covariates during single group voxel-wise VBM, we performed a separate analysis of the similar design using the first three PCs. It reveals a cluster of GM volume in the linear relationship with the PC2 component that topographically involves the superior parietal lobule and precuneus on the left hemisphere (Fig 4).

## Discussion

This study focused on the volumetric structural abnormalities of the cortical and subcortical GM and its clinical correlates in children with AS. Although quantitative neuroimaging approaches have been already applied to study WM abnormalities in AS, the whole-brain investigation of possible GM changes has not been performed yet. Our FSL-VBM analysis demonstrates bilateral GM volume loss in AS compared to control children in the basal ganglia (BG), limbic structures, insular and orbitofrontal cortices. Voxel-wise correlation analysis with three principal components, which represent and explain the variation of the complex neurodevelopmental and neuropsychological data, revealed a significant relationship between GM volume in the superior parietal lobule and precuneus on the left hemisphere. The anatomical distribution of our findings provides an important morphological underpinning for clinical and neurobehavioral symptoms in children with AS.

In agreement with previous scientific reports, the consistent clinical features of our AS patients represent severe intellectual delay, movement or balance disorder, behavioral uniqueness, and speech impairment [11,38]. The majority of our children with AS manifest with seizure disorder with the onset before 3 years of age, which is a consistent finding in Angelman research [11,24]. Although, these clinical manifestations can be attributed to the disruption of the UBE3A gene, regardless of the underlying molecular mechanism, the AS children with deletions have more severe symptoms, probably due to haploinsufficiency of genes adjacent to UBE3A [9]. They are more likely to have seizures and more severe language impairment compared to those with UPD, UBE3A mutations or imprinting defects [9]. In this study, we included children with AS due to deletion of the maternally inherited UBE3A gene, although our AS cohort represents also minor numbers of AS children caused by UPD, UBE3A mutations, and imprinting defects.

Animal studies have shown that the maternal deficient heterozygous UBE3A knockout mice exhibited reduced brain weight [12]. In agreement with these findings, our SIENAX output showed smaller global WM and GM volumes in AS patients compare to controls. Taking into account that microcephaly is a common feature of AS children, we analyzed total brain tissue volume normalised for each subject head size (SIENAX pipeline). Care was also taken to include global WM, age, and gender as confounding covariates for voxel-wise group comparisons. Between-group exploratory VBM analysis revealed a profound GM volume loss in AS patients in the bilateral BG, largely involving dorsal and, with less extend, ventral striatum. The two huge clusters of the left and right hemisphere show topographical symmetry in the striatal level, but then they extend more prominently to the frontal orbital cortex on the right hemisphere and to the temporal-mesial structures in the left hemisphere largely encompassing the left amygdala (Fig 3).

Nowadays, the BG circuitry is gaining more attention in AS research, first, because of its role in the motor and balance control, and second, because of its contribution to non-motor domains of AS phenotype [8]. From the traditional point of view, the functional role of the striatum is described in the context of the neuroanatomical and functional “loops” of BG, which plays a key role in the modulation of motor functions and motor control [39]. It is well known that the movement or balance disorder is one of the most consistent findings in AS and characterized by uncoordinated movements with broad-based gait, rigid lower limbs on ambulation with trembling or jerky motions of the limbs, and disturbed sense of equilibrium especially during an upright posture [10]. Recently, it has been shown that cerebellar function is only slightly impaired in the UBE3A mouse model of AS, thus enhancing the contribution of the BG in the locomotor deficits of AS mice [40].

Furthermore, the motor impairments in AS, such as non-epileptic tremor, which is presented in the majority of our patients, can be ameliorated by Levodopa treatment [41]. These facts signify the attribution of abnormal dopaminergic signaling in the striatum of the AS and has been even verified by direct measurement of dopamine using fast-scan cyclic voltammetry [42]. Recent animal studies demonstrated abnormal nigrostriatal pathway in UBE3A knockout mice and marked loss of UBE3A expression in the striatum [43,44]. Hayrapetyan et al. [7] showed a selective deficit in the instrumental conditioning (a striatum-dependent task) in the UBE3A deficient mouse model, thus suggesting a specific impairment in glutamatergic transmission in the associative cortico-striatal circuit in AS. Taking into account the pivotal contribution of the BG in the pathophysiology of AS, we suppose that severe bilateral GM loss in the striatum, as shown in our study, may be responsible for abnormal movements, tremor, and dyscoordination presented in the individuals with AS.

Furthermore, clinical and experimental data have been gathered to support the idea that the BG could be involved in the interruption of epileptic seizures and modulation of their occurrence [45]. It has been shown that patients with refractory epilepsy and drug-resistant complex partial status epilepticus exhibit decreased [18F] Fluoro-L-Dopa uptake in the caudate and putamen nuclei during ictal discharges [46]. Besides, the most of our patients present with epileptic seizures of longer duration and it seems that striatal dopaminergic activity deficit with the consequent inability of BG to interrupt seizures might play a key role in the genesis of prolonged seizures [46].

The latest advances in modern neuroimaging techniques, involving diffusion tensor imaging (DTI) and resting state functional connectivity (rest-fMRI) studies of the human striatum substantially support the growing evidence in the cognitive contribution of the striatum. Neuroanatomical studies have described multiple functional loops between striatum and the prefrontal cortex, thus linking striatum to cognitive and executive functioning. Present-day consideration is that the ventral striatum mediates reward processing [47], while the dorsal striatum is involved in cognitive functions, such as working memory, executive function, strategic planning, task switching, rule learning, effective inhibition of conflicting information and certain aspects of language processing [48]. Therefore, striatal GM loss might be also related to cognitive and psychomotor delay in AS due to aberrant connectivity between basal ganglia and prefrontal cortex, which is essential for mediating high-order executive function and top-down regulation of cognition.

In AS the developmental delay is usually evident within the first year of life, with hanging in the attainment of gross motor, fine motor, receptive language, expressive language, and social skills. The ceiling for psychomotor development is around the 24–30 months range [49,50] and remains severe at the functional level. In agreement with this, our patients had reached and hanged over the median developmental age of 15.6 ± 5.1 months on the Bayley III Cognitive Scale, varying from 7 to 22 months. Expressive language was very poor or even absent and was impaired to a greater extent than receptive language. The median developmental age on the Bayley III Language Scale was 11.1 ± 4.5 and 15.8 ± 5.3 months for expressive and receptive communication, respectively (Table 1).

The next consistent feature of AS is a behavioral uniqueness with the combination of frequent laughter and smiling, apparent happy demeanor, easily excitable personality often with hand flapping or waving movements, hyper-motor behavior including restlessness or distractibility [38]. We presume that our findings of GM volume loss in the OFC and temporal-mesial structures may support neuroanatomical signature of behavior peculiarities in AS. The OFC is a subdivision of the prefrontal cortex with reciprocal connections with neuroanatomical structures, such as the hippocampal formation, amygdala, ventral striatum, anterior cingulate, hypothalamus and medial temporal areas [51]. The OFC has been associated with emotional and executive processing, decision making, adaptive and reward-guided behavior [51,52]. In addition, lesion-based studies observed the persistent association between OFC damage with socioemotional disinhibition and executive dysfunctions [53].

Besides, the amygdala is well fortified as a key player in emotion, motivation, fearful and reward processing [54]. Both amygdala and hippocampus are derived from the telencephalon and both constitute largely to the limbic system. On the contrary to the hippocampus, the amygdala has not gained much interest in AS research yet, although the functional role of amygdala might relate to some core clinical features of AS, including altered emotional behavior and pathological laughter [55]. Amygdala receives information about the external environment from the sensory thalamus, sensory cortices and reciprocally connected with cortical regions, in particularly with OFC and midline prefrontal cortices, the hippocampus, as well as sensory association areas [54]. We think that GM loss in the amygdala of individuals with AS may provide a plausible interpretation for their affective lability, presuming that due to amygdala-prefrontal cortex dysregulation the affect may be mood incongruent or may be greatly in excess of the associated mood changes [56].

The clinical and neurobehavioral manifestations of AS are broad and probably involve multiple interconnected neuroanatomical subunits. As a result, it is not surprising that the voxel-wise correlation analyses with each single neurodevelopmental and neuropsychological measurements revealed no relationship with GM volume. Taking into account that several neurodevelopmental and neuropsychological test scores are inter-correlated, the PCA analysis may represent the optimal choice to extract the important information into low-dimensional uncorrelated PCs before applying voxel-wise correlation analysis. As a result, we revealed a small cluster of GM in a strong relationship with PC2 in the superior parietal lobule and precuneus on the left hemisphere (Fig 4). The PC2 is mostly condensed the GMFM tests scores that represent motor skills and gross motor functions, hence, we speculate that the distribution of GM changes on these regions might be related to optic ataxia and divergent spatial representation of the motor movements in AS [57]. However, the exact clinical and neurobehavioral correlates of this finding are hard to conclude and necessitate future studies.

It is a great importance the understanding of the neuroanatomical contribution to the developmental and cognitive functioning in AS, thus, helping to link molecular pathophysiology to clinical phenotype. In our study, we demonstrate novel findings of widespread neuropathological changes in the cortical and subcortical GM in AS that can be plausibly related to the symptoms and signs of the disease. Nevertheless, there are some limitations to this study. First, the sample size is limited and requires further age-related stratification. Furthermore, the AS population included in our study may not be representative of individuals with other genetic subtypes of AS due to UPD, UBE3A mutations or imprinting defects. Next, AS children and the control subjects have contrasting abilities and performance in different cognitive domains, which may appear important confounding factor. However, taking into account that AS is a rare disorder, in our study, we were able to reach a relative larger cohort of AS children due to molecularly confirmed maternal deletion of the chromosome 15q11.2–13.

In summary, we determine for the first time regional specific cortical and subcortical GM alterations in AS. The FSL-VBM analysis demonstrates bilateral GM volume loss in the BG, limbic structures, insular and orbitofrontal cortices in AS compared to control children. The distribution of GM changes is consistent with several clinical and neurobehavioral features of the disease. At present, it is hard to determine the primary link between the molecular pathophysiology and the regional-selective GM volume loss. Further studies are needed to establish the molecular relevance of our findings in patients with AS.
